# Posterior cruciate ligament repair seems safe with low failure rates but more high level evidence is needed: a systematic review

**DOI:** 10.1186/s40634-023-00605-z

**Published:** 2023-04-26

**Authors:** Jasper Vandenrijt, Sofie Callenaere, Dries Van der Auwera, Jozef Michielsen, Pieter Van Dyck, Christiaan H. W. Heusdens

**Affiliations:** 1 Orthopaedics, Antwerp University Hospital, Drie Eikenstraat 655, Edegem, 2650 Belgium; 2 Faculty of Medicine and Health Sciences, University of Antwerp, Universiteitsplein 1, Wilrijk, 2610 Belgium; 3 Department of Radiology, Antwerp University Hospital, Drie Eikenstraat 655, Edegem, 2650 Belgium

**Keywords:** Posterior cruciate ligament, PCL, Repair, Clinical outcomes, Systematic review

## Abstract

**Purpose:**

To discuss recent literature on posterior cruciate ligament (PCL) repair and report on the clinical and radiological outcomes.

**Methods:**

A systematic review was conducted according to the PRISMA guidelines. In August 2022, three databases (PubMed, Scopus, and Cochrane Library) were searched for studies on PCL repair by two independent reviewers. Articles published between January 2000 and August 2022 focussing on the clinical and/or radiological outcomes, following PCL repair, were included. Patient demographic data, clinical evaluations, patient‑reported outcome measures, post-operative complications and radiological outcomes were extracted.

**Results:**

Nine studies met the inclusion criteria, covering 226 patients with a mean age ranging from 22.4 to 38.8 years and mean follow-up periods ranging from 14 to 78.6 months. Seven studies (77.8%) were level IV and two studies (22.2%) were level III. Arthroscopic PCL repair was performed in four studies (44.4%) while the remaining five studies (55.6%) described open PCL repair. In four studies (44.4%) additional suture augmentation was applied. Arthrofibrosis affected a combined total of 24 patients (11.7%; range 0–21.0%) making it the most common complication and the overall failure rate was 5.6%, ranging from 0 to 15.8%. Two studies (22.2%) performed post-operative MRI and confirmed PCL healing.

**Conclusion:**

This systematic review indicates that PCL repair can be a safe procedure with an overall failure rate of 5.6%, ranging from 0% to 15.8%. However, more high quality research is necessary before widespread clinical implementation is warranted.

**Level of Evidence:**

IV.

**Supplementary Information:**

The online version contains supplementary material available at 10.1186/s40634-023-00605-z.

## Introduction

Injuries to the PCL are rare and less common compared to ACL ruptures. Depending on the clinical setting in which data is collected, the reported incidence of PCL injuries ranges from 1 to 40% of all acute knee injuries [11, 14]. High velocity trauma (i.e., road accidents) resulting in so‑called “dashboard injuries” are the main cause of PCL tears [10, 23, 51]. PCL ruptures are rarely isolated, and often occur concurrently with other knee injuries in a multiligament knee injury (MLKI) setting [10, 36, 44].

The PCL is known to possess an intrinsic healing capacity [29, 52]. Due to the ability to self-heal, non-operative management is recommended for grade I or II PCL ruptures according to our current standard of care model [2, 17, 48]. However, there is a risk that healing occurs in a lax or attenuated manner [31, 44] and studies have shown that non‑operatively treated PCL ruptures increased the risk of developing osteoarthritis in the PCL deficient knee, compared to the contralateral knee [3, 58].

Operative management is preferred in grade III PCL injuries or in physically active patients with severe symptoms. Throughout the 1980s and 1990s, primary PCL repair was a commonly practiced treatment for PCL ruptures [7, 28, 57]. Despite good initial results, long term results were less favourable [47, 49, 60]. Richter et al. reported mediocre results of primary repair in one third of the patients and 28% was unable to return to their preinjury activity level [49].

However, it should be noted that post-operative care was fundamentally different, as these early primary repairs were often followed by cast immobilization, instead of immediate intensive physiotherapy to restore range of motion (ROM) [43, 54, 57]. The inferior results ultimately led to the abandonment of primary PCL repair in favour of cruciate reconstruction [12, 38].

Although, reconstruction of a torn PCL is considered the current gold standard as surgical approach, a lot of controversy remains on which technique to use (single bundle vs. double bundle; transtibial vs. tibial inlay) and which graft material to use [36, 44, 61]. Additionally, results of reconstruction remain inconclusive as normal knee stability is not restored and patients are still at an increased risk of developing osteoarthritis [33, 52, 56].

In recent years, primary PCL repair is starting to resurge, owing to the promising results of innovative ACL repair techniques [9, 20, 34, 41]. Over the last few years a number of case series and cohort studies have been published reporting on primary PCL repair. The purpose of this article is to systematically review recent literature evaluating primary PCL repair interventions and the clinical and radiological outcomes to gain a more thorough understanding regarding PCL repair. This systematic review could provide important insights for orthopaedic surgeons who are interested in adopting a novel PCL repair technique in their arsenal.

## Methods

### Search strategy

A systematic review of recently published literature was conducted according to the Preferred Reporting Items for Systematic Reviews and Meta-Analyses (PRISMA) guidelines concerning the treatment of posterior cruciate ligament ruptures with a surgical repair technique [45].

To identify published studies on PCL repair, a literature search was performed by two independent authors (S.C. and D.V.D.A.) across three electronic databases (PubMed, Scopus, and Cochrane Library). Search terms included “Posterior Cruciate Ligament”, “Lesion” and “Primary Repair”. An example of the PubMed search strategy can be found in the Additional file 1. The search covered articles published between January 2000 until August 2022. Duplicates were removed and the identified articles were screened by title and abstract. Subsequently, full texts of the remaining articles were reviewed against the eligibility criteria. Inclusion criteria were defined as: (i) patients undergoing primary PCL repair, (ii) minimal clinical and/or radiological post-operative follow-up of 12 months (iii) written in English, French or German and (iv) studies with a level of evidence (LOE) of IV or more. Exclusion criteria were: (i) pre‑clinical studies, (ii) case studies with a sample size of 3 or less, (iii) bony avulsion fractures, (iv) studies focussing primarily on a paediatric population (< 18 years), (v) studies published before 2000 and (vi) studies from the same research group with overlapping study samples. In that case the study with the smallest sample size or shortest follow-up will be excluded.

Finally, to identify missed publications, the reference sections of the included articles were checked. Inconsistencies between the authors were resolved by consensus or the decision of a third independent reviewer (J.V.).

### Data extraction

Data extraction was performed subsequently by three independent reviewers (S.C., D.V.D.A. and J.V.). Extracted data from each full-text article included study characteristics: lead author, publication year, sample size, age, gender, mean time of follow-up, time to surgery, tear location and type of surgery. The collected outcome measures consisted of failure rate, complications and knee stability. As clinical outcome scores, the Lysholm score [4], International Knee Documentation Committee (IKDC) score [22], both pre-injury and post-operative Tegner score [59], Knee Injury and Osteoarthritis Outcome Scale (KOOS) [50] and Visual Analogue Scale (VAS) for pain score and patient satisfaction [30, 64] were considered relevant for extraction. Degenerative changes and PCL healing were assessed by extracting radiological data. All data were collected in Excel 2016 (Microsoft Corp., Redmond, WA, USA).

The LOE was determined by applying the modified criteria of Oxford Centre for Evidence-Based Medicine Working Group [39, 68]. Each study was assigned a LOE according to study design [39]. The methodological quality of the included studies was estimated using the Methodological Index for Non-randomized Studies (MINORS) instrument. Only the first eight items were used which are specifically developed for non-comparative studies. Each individual item is scored (0 to 2) and the summation of these items will make up the MINORS score. For non-comparative studies the maximal score is 16 [55].

### Data analysis

A pooled analysis of the outcome data was deemed inappropriate due to the inclusion of nonrandomised studies. This resulted in a collection of studies with an overall low LOE and ample risk of bias. Additionally, there is a high degree of heterogeneity across the studies as different surgery techniques were described and follow-up periods varied considerably. Therefore, the outcome data will be reported as a narrative synthesis. The included studies will be categorised based on surgical intervention (open or arthroscopic) and the means of the outcome measures including knee stability and Patient-Reported Outcome Scores (PROMs) will be presented and discussed as ranges rather than pooled means.

## Results

### Literature search

**Fig. 1:**
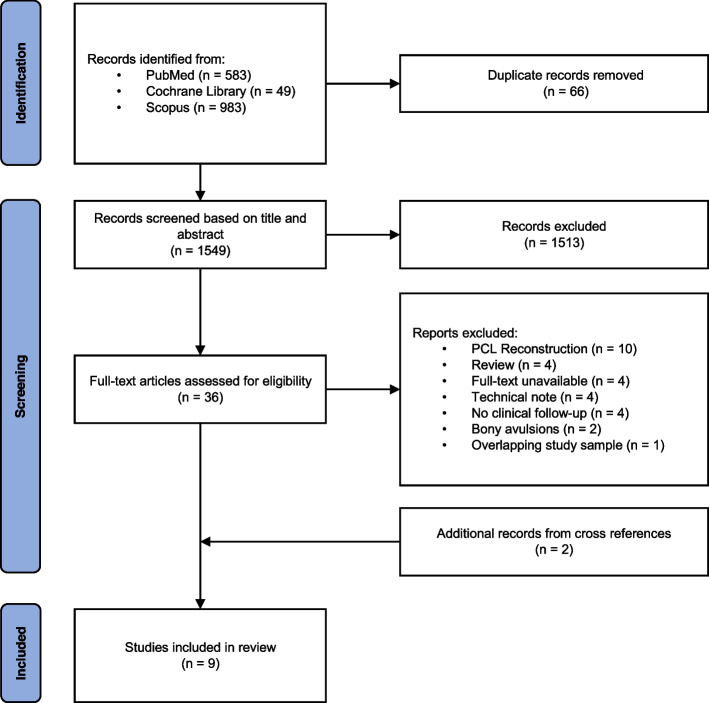
PRISMA flowchart describing the inclusion process of relevant studies

The literature search identified 1615 articles from three online databases. A flowchart of the selection process is presented in Fig. 1. After removal of 66 duplicates, titles and abstracts were screened by the two independent reviewers, leaving 36 articles for full-text review. Seven studies were considered eligible for this systematic review and after reviewing the reference lists, two additional studies were identified. In total there were nine studies that described the results of primary PCL repair [18, 25, 27, 35, 42, 43, 53, 62, 65].

### Study characteristics

The study characteristics of all included studies are presented in Table 1. Considering study design, there were no level I or level II studies identified, two studies were level III cohort studies (22.2%) [53, 62] and seven studies were level IV case series (77.8%) [18, 25, 27, 35, 42, 43, 65]. Furthermore, six studies used a retrospective approach (66.7%) and three studies a prospective (33.3%). The methodological quality scores, as determined using the MINORS instrument, ranged between 8 and 12, with a mean score of 10.1 out of 16. The main weaknesses of the included studies were a lack of sample size calculations, blinding and reporting dropouts.

**Table 1:** Study characteristics of all included studies

Author	Year	LOE	Prospective/retrospective	Study design	Surgery type		MINROS								
					**Open/arthroscopic**	**Suture augmentation**	**1**	**2**	**3**	**4**	**5**	**6**	**7**	**8**	**Total**
Heitmann et al	2019	IV	Prospective	Case series	Open	Yes	2	2	2	2	1	1	1	1	12
Hopper et al	2021	IV	Prospective	Case series	Arthroscopic	Yes	2	2	2	2	0	2	1	1	12
Hua et al	2016	IV	Retrospective	Case series	Open	No	2	2	1	2	0	2	1	0	10
Kohl et al	2015	IV	Prospective	Case series	Open	No	2	2	2	2	0	1	0	0	9
Otto et al	2020	IV	Retrospective	Case series	Arthroscopic (11/14)	Yes	2	1	1	2	0	1	1	2	10
Owens et al	2007	IV	Retrospective	Case series	Open	No	2	2	1	2	0	2	1	1	11
Shirakura et al	2001	III	Retrospective	Cohort	Open	No	2	2	1	1	0	2	0	0	8
Vermeijden et al	2020	III	Retrospective	Cohort	Arthroscopic	Yes	2	2	1	1	0	2	2	0	10
Wheatley et al	2002	IV	Retrospective	Case series	Arthroscopic	No	2	2	1	2	0	2	0	0	9

*LOE* Level of evidence, *MINORS* Methodological Index for Non-randomized Studies

The 8 items of the MINORS; 0 = not reported; 1 = reported but inadequate; 2 = reported and adequate:

1. A clearly stated aim

2. Inclusion of consecutive patients

3. Prospective collection of data

4. Unbiased assessment of the study end point

5. Follow-up period appropriate to the aim of the study

6. Loss to follow-up less than 5%

8. Prospective calculation of the study size

### PCL repair technique

Arthroscopic primary PCL repair was performed in four studies (44.4%) [25, 42, 62, 65], with three of these studies (35.7%) applying additional suture augmentation, consisting of an *Internal*Brace™ (Arthrex, Naples, Florida) [24], to reinforce the healing ligament [25, 42, 62]. Femoral fixation was achieved using transosseous tunnels in two studies (22.2%) [25, 65], while one study (11.1%) used the suture anchor technique to reattach the PCL to its femoral footprint [62]. Otto et al. [42] used suture anchors for proximal tears while transosseous tunnels were used for mid-substance and distal tears.

Open primary PCL repair was described by five studies (55.6%) [18, 27, 35, 43, 53]. Only one of these studies (11.1%) performed additional augmentation. Heitmann et al. [18] used a ligament bracing technique similar to the suture augmentation method described in arthroscopic PCL repair studies [25, 42, 62]. In the remaining four studies (44.4%) open PCL repair was performed without using additional suture augmentation [27, 35, 43, 53]. All open PCL repair studies used transosseous tunnels to reattach the torn PCL, with two studies creating multiple tunnels to form a bone bridge [27, 43].

### Patient demographics

All studies combined covered a total of 226 patients (230 knees), which included 165 (73.0%) males and 61 (27.0%) females (Table 2). Sample sizes ranged from 11 to 69 patients with a median of 19 patients per study. The mean age of the patients included in the individual studies ranged from 22.4 to 38.8 years and the absolute age ranged from 13 to 75 years. On average, time between injury and surgery ranged from 7.3 days to 17 days and post-operative follow‑up ranged from 14 to 78.6 months. All studies but one [53] included MLKI. Most ruptures (75.0%) were caused by high velocity traumas, i.e., motor vehicle accidents. Low-velocity traumas, i.e., sports accidents or falls, were less common accounting for 25.0%. As depicted in Table 3, four studies reported on tear location. The majority of the repaired PCL ruptures were located in the in proximal third (54.0%) of the ligament while ruptures in the middle (19.0%) or distal (27.0%) third were less common.

**Table 2:** Patient demographics

Author	Sample size	Agea	Gender		Follow-up (months)^a^	MLKI	Cause	Time to surgery (days)^a^
			Male	Female				
Heitmann et al	69	34.2 (18–60)	49	20	14 (12–18)	Yes (dislocation)		7.3
Hopper et al	16	37 (19–57)	16	0	48 (24–66)	Yes (5/16)		
Hua et al	17 (18 knees)	38.8 (19–62)	10	7	57.6 (29–88)	Yes (dislocation)	11 HVT; 6 LVT	5–10
Kohl et al	35	33.4 (17.4–55.6)	26	9	26 (12–42)	Yes (dislocation)	35 HVT	< 2^b^
Otto et al	14	37.4 (16–75)	8	6	19.9 (12–35)	Yes		10.2
Owens et al	25 (28 knees)	35.2 (17–67)	19	6	48 (13–82)	Yes (dislocation)	19 HVT; 6 LVT	17 (1–101)
Shirakura et al	20	26.4 (13–49)	17	3	79 (62–120)	No	14 HVT; 6 LVT	< 14^b^
Vermeijden et al	19		12	7	> 6	Yes		15
Wheatley et al	11	22.4 (15–45)	8	3	51.4 (13.4–127.9)	Yes	2 HVT; 9 LVT	16.2 (0–48)

*MLKI* multi ligament knee injury, *LVT* Low velocity trauma, *HVT* High velocity trauma

^a^Data are presented as Mean (Range)

^b^The time frame in which surgery was carried out

**Table 3:** Tear location

Author	Tear location				
	**Proximal avulsion**	**Proximal**	**Medial**	**Distal**	**Distal avulsion**
**Open PCL repair**					
Heitmann et al	*Unspecified*				
Hua et al	*Unspecified*				
Kohl et al	*Unspecified*				
Owens et al	*Unspecified*				
Shirakura et al	0	4	6	10	0
**Arthroscopic PCL repair**					
Hopper et al	*Unspecified*				
Otto et al	0	5	6	2	1
Vermeijden et al.^a^	1	13	0	3	1
Wheatley et al	11	0	0	0	0

^a^Vermeijden et al. included 1 bony avulsion

### Outcome scores

An overview of the clinical outcomes, collected at final follow-up, is presented in Table 4. Frequently reported PROMs were the Lysholm, IKDC and Tegner scores. The Lysholm score was utilised by six studies (66.7%) and ranged between 69.1 and 95.4 [18, 27, 35, 42, 43, 65]. Four studies using an open PCL repair approach reported mean Lysholm scores ranging between 81.0 and 90.8 [18, 27, 35, 43]. Considering the studies on arthroscopic PCL repair, Wheatley et al. demonstrated a mean post-operative Lysholm score of 95.4, whereas Otto et al. reported a mean Lysholm score of 69.1 [42, 65]. Pre- and post-operative scores were not compared in the included studies.

**Table 4:** Clinical evaluation outcomes^a^

Author	Lysholm	IKDC (%)	Tegner		KOOS					VAS		Posterior knee translation		
													**Side to side difference**	
			**Pre-injury**	**Post-injury**	**Pain**	**Sym**	**ADL**	**QoL**	**Sports**	**Pain**	**Satisfaction**	**PDT**^c^	**Stress radiograph**	**KT arthrometer**
**Open PCL repair**														
Heitmann et al	81, SD ± 15.5	75.5, SD ± 14.5	6 (3–8)^b^	5 (1–7)^b^									2.9 mm, SD ± 2.1	
Hua et al	87.5, SD ± 7.7 (71–95)		5.59, SD ± 1.4 (3–9)	3.35, SD ± 1.7 (1–6)						2.43, SD ± 0.89 (1–4.5)	7.98, SD ± 1.12 (5.1–9.2)			0.8 mm, SD ± 0.4 (0.1 – 1.2)
Kohl et al	90.8 (81–95)		6.9 (5–10)	4.8 (3–9)							8.7 (5–10)			2.5 mm
Owens et al	89 (68–100)		5.6 (3–10)	4.4 (1–9)										
Shirakura et al														
**Arthroscopic PCL repair**														
Hopper et al					87	75.5	93	54.2	69.6	0.8				
Otto et al	69.1, SD ± 16.6	68.9, SD ± 18.1		4^b^									5.5 mm, SD ± 4.1	
Vermeijden et al														
Wheatley et al	95.4 (90–100)											5 Neg. 6 Grade 1	2.6 mm (0–6)	

*Sym* Symptoms, *ADL* daily living activities, *QoL* Quality of Life, *PDT* Posterior drawer test

^a^Data are presented as Mean, ± Standard Deviation and Range for studies reporting these measures

^b^Data reported as Median

^c^PDT, Posterior drawer test; Neg, negative: 0-5 mm, Grade 1: 5-10 mm, Grade 2: > 10 mm

Four studies reported IKDC scores [18, 35, 42, 65]. Among the studies utilising open PCL repair, Heitmann et al. reported an IKDC score of 75.5% after open PCL repair with suture augmentation [18] and in the case series of Kohl et al. the IKDC scores were rated nearly normal in 29 patients (83%) and abnormal in 6 patients (17%) [35]. Considering the studies on arthroscopic PCL repair, Otto et al. [42] reported a mean IKDC score of 68.9% and Wheatley et al. [65] described eight patients (73%) with normal and three patients (27%) with nearly normal IKDC scores.

The Tegner activity scoring system was utilized by five studies, with four studies reporting both pre-injury and post‑operative scores [18, 27, 35, 42, 43]. The mean difference in Tegner score, pre-injury to post-operative, ranged between ‑1 to -2.24 in patients treated with open PCL repair, with two studies reporting a statistically significant decrease in Tegner activity score [18, 27]. Furthermore, following arthroscopic PCL repair, Otto et al. observed a median post-operative Tegner score of 4 [42].

Knee stability based on side-to-side posterior translation was reported by five studies (55.6%), of which three studies applied open PCL repair [18, 27, 35] and two used an arthroscopic approach [42, 65]. A mean side-to-side difference of 2.9 mm was observed by Heitmann et al. using stress radiographs [18]. In addition, Hua et al. [27] and Kohl et [35] reported a posterior translation of 0.8 mm and 2.5 mm, respectively, using KT arthrometers [27, 35]. Studies focusing on arthroscopic PCL repair reported differences in side-to-side posterior translations ranging from 2.6 to 5.5 mm based on stress radiographs [42, 65].

### Imaging

Preoperatively, PCL ruptures were often diagnosed using MR imaging [18, 35, 42, 43, 62, 65]. Two studies, comprising a total of 25 patients, assessed post-operative MRI scans to evaluate the appearance and healing status of the repaired PCL [42, 65]. Otto et al. [42] investigated PCL overall continuity, signal intensity and morphology to assess PCL healing. At a mean follow-up of 19.9 months (range 12–35), the authors confirmed healing of the repaired PCL [42]. While not describing MRI parameters or image interpretation methods, Wheatley et al. [65] reported PCL healing on MRI.

In one study, Shirakura et al. [53] used radiographs to assess the development of degenerative changes by applying the Fairbank’s criteria (grade 0 to 4) relative to the contralateral leg. In a study sample of 20 patients, three patients displayed degenerative changes after a mean follow-up of 6 years and 7 months [53].

### Complications

Post-operative complications were discussed in eight of the nine studies (88.9%), covering a combined total of 206 patients. A summary of the complications can be found in Table 5.

**Table 5:** Complications and failures related to PCL repair

Author	Failures		Complications	
	**Instability**	**Re-rupture**	**Arthrofibrosis**	**Other**
**Open PCL repair**				
Heitmann et al	8.7% (6/69)^a^		11.6% (8/69)	1.4% (1/69): Compartment syndrome 1.4% (1/69): Infection
Hua et al			17.6% (3/17) (18 knees)	5.9% (1/17) (18 knees): Infection 5.9% (1/17) (18 knees): Fat liquefaction
Kohl et al			5.7% (2/35)	
Owens et al			20% (5/25) (28 knees)	4% (1 (2 knees) of 25 (28 knees)): Heterotopic ossification 8% (2/25 (28 knees)): Stitch granuloma
Shirakura et al				Unknown^b^
**Arthroscopic PCL repair**				
Hopper et al		6.3% (1/16)		
Otto et al	14.3% (2/14)		7.1% (1/14)	
Vermeijden et al	15.8% (3/19)		21.1% (4/19)	
Wheatley et al			9.1% (1/11)	

^a^Of the six failures, four were related to the ACL

^b^The authors did not clearly state whether or not any complications occurred

Post-operative stiffness owing to arthrofibrosis was the most common complication affecting a combined total of 24 patients (11.7%; range 0–21.0%) [18, 27, 35, 42, 43, 62, 65]. Open PCL repair resulted in a combined arthrofibrosis rate of 12.3% (range 5.7–20.0%). Of the patients treated with arthroscopic PCL repair, 10.0% (range 0–21.1%) developed arthrofibrosis. Management of this complication consisted of physical therapy, manipulation under anaesthesia and/or surgical lysis of adhesions.

Two patients of two separate studies suffered from wound infection, which was treated with antibiotics and debridement [18, 27].

One case of compartment syndrome was reported by Heitmann et al., which required revision surgery using a fasciotomy [18]. Furthermore, in the case series of Owens et al., there was one patient of the 25 (4.0%) who developed heterotopic ossification in both knees and two patients (8.0%) with stich granuloma [43]. At last, out of 16 patients Hua et al. encountered one case (5.9%) with fat liquefaction [27].

Four studies (44.4%) documented cases of PCL repair failure, defined as either a re-rupture [25] or abnormal laxity [18, 42, 62]. Hopper et al. [25] encountered one re-rupture in a sample size of 16 (6.3%) which was surgically treated using PCL reconstruction three years after the initial repair. Three clinical failures (15.8%), due to insufficient knee stability, were reported by Vermeijden et al. [62], two of which received subsequent PCL reconstruction while the remaining patient was treated conservatively. Six clinical failures (8.7%) were observed by Heitmann et al. within two years of follow-up [18], four patients were treated with subsequent ACL reconstruction and two patients required multiligament reconstruction. Otto et al. [42] reported two clinical failures (14.3%) characterised by 2 + posterior translation in a sample of 14 patients. Based on these results a combined failure rate of 5.8% (range 0–15.8%) was calculated. For open and arthroscopic PCL repair, the failure rates were or 4.1% (range 0–8.7%) and 10.0% (range 0–15.8%), respectively.

### Post-operative rehabilitation

Seven studies (77.8%) provided a rehabilitation protocol [18, 25, 27, 35, 42, 43, 53]. An in depth overview of the rehabilitation programs used in the included studies is presented in Table 6. In general, hinged knee braces were used immediately after surgery to limit ROM to 90° flexion or fixate the knee in full extension. During the first post‑operative days, the rehabilitation programs focussed on regaining quadriceps strength by performing isometric muscle contractions and ROM exercises were also performed up to 90° flexion. After four to six weeks, ROM was gradually increased to full ROM. In addition, closed-chain exercises were initiated, allowing hamstring co‑contraction. Open-chain exercises were usually not started before three months post-operative.

**Table 6:** Detailed overview of the post-operative rehabilitation programs

Author	Weeks 1–4	Weeks 5—11	Months 3–6	Months 6 +	Brace
**Open PCL repair**					
Heitmann et al	PWB for 6 weeks, brace limiting ROM to 0/90°	Week 6, brace no longer limits ROM	3 months, brace removed	NR	Stabilizing braces
Hua et al	first week brace locked in 30°; isometric quadriceps exercises 1-day post-surgery; after 1 week passive ROM	After 4 weeks, non‑weight bearing activities and passive ROM exercises increasing flexion to 120°	3 months, closed chain exercises and hamstring co-contractions; 4–5 months, open-chain exercises, walking, PWB	6 months, progressive resistive exercises; 7 months, walking without crutches, FWB	Hinged knee brace
Kohl et al	PWB (15 kg), brace limiting ROM to 90° 0–1 week, activation extensors; Week 3; flexors, ROM and proprioception exercises	Week 6, brace removed; progression to full active ROM and FWB	3 months, possible to start open chain exercises	NR	Hinged knee brace
Owens et al	Day 1, passive ROM 0° to 30°; By day 10, reaching a passive ROM of 90°; Week 1–4, Quadriceps strengthening	Week 4, hamstring co-contraction; progressing ROM to 120° and non‑weight bearing using crutches until week 8; week 8–12 PWB using crutches	3 months, FWB without crutches and closed-chain exercises and hamstring co-contraction; 4 months, open-chain exercises	Unrestricted activities	Hinged knee brace
Shirakura et al	first 4 weeks, toe-to-groin cast; isometric quadriceps exercises; week 3, weight bearing	Cast removal and start of isotonic and ROM exercises	NR	NR	Toe-to-groin cast
**Arthroscopic PCL repair**					
Hopper et al	First 2 weeks, FWB using crutches allowed; early ROM	NR	NR	NR	NR
Otto et al	week 1–6, PWB up to 20 kg, passive mobilisation, brace limiting ROM to 90°	week 7, gradual progression to FWB, knee only during daytime	3 months, walking, cycling, swimming;	6 months, brace removed, jogging or sport specific exercises; 9–12 months, return to sport	Knee brace with posterior tibia support
Vermeijden et al	*Unspecified*				
Wheatley et al	*Unspecified*				

*PWB* Partial weight bearing, *FWB* Full weight bearing, *ROM* Range of motion

## Discussion

The purpose of this systematic review was to report on the clinical and radiological outcomes of PCL repair. In total, our search retrieved nine papers reporting on the outcomes of PCL repair, covering 226 patients. Overall, PCL repair can be a safe and effective treatment option with a combined failure rate of 5.8% (range 0–15.8%) and with only one study revealing PROMs < 80% of the maximal score. The available literature showed that PCL repair has gained attention in recent years, but the current research remains restricted to small case series providing low level evidence.

To date PCL reconstruction is considered the standard operative treatment for grade 3 PCL ruptures. Comparing PCL repair to PCL reconstruction is difficult as none of the included studies directly compared both techniques. The present systematic review demonstrates side-to-side differences in laxity ranging from 0.8 – 2.5 mm and 2.6 – 5.5 mm based on KT arthrometer and Stress radiographs, respectively. This appears to be in line with results reported in a recent systematic review focusing on single bundle and double bundle PCL reconstruction by Chahla et al. [6]. The authors reported KT arthrometer based side-to-side differences in laxity ranging from 1.91 to 4.5 mm and 1.78 to 4.3 mm for single bundle and double bundle reconstruction, respectively. Based on Telos stress radiographs side-to-side differences ranged from 2.56 to 5.6 mm and 2.36 to 4.9 mm for single bundle and double bundle reconstruction, respectively [6].

Throughout the included studies, multiple surgical repair procedures were proposed. Most of these studies described a surgical approach that used the transosseous tunnel technique to either direct the PCL to its femoral footprint or to fixate the additional suture augmentation [18, 25, 27, 35, 42, 43, 53, 65]. Suture anchors were used in two studies for the femoral fixation of proximal tears [42, 62]. Interestingly, all studies published since 2019 applied an internal brace as suture tape augmentation [18, 25, 42, 62], a technique which is also used to repair ACL ruptures [5, 19, 20, 67]. This highlight how the promising results of modern ACL repair have sparked a renewed interest in PCL repair as well. Similar to ACL repair, PCL repair has several theoretical benefits, compared to reconstruction. Firstly, retention of the native ligament. This could be important given the variety of mechanoreceptors that have been identified in the PCL which aid knee stability [1, 32]. A recent meta-analysis reported loss of proprioception in PCL deficient knees after PCL reconstruction [69]. By restoring the native anatomy, the proprioceptive function of the PCL is preserved [63]. Secondly, there is no need for graft harvesting which means no donor site morbidity [63]. Thirdly, when adding a secondary stabilizer, i.e., an internal brace, the healing ligament is protected whilst early mobilization is permitted. These advantages could ultimately reduce the rehabilitation period.

Nevertheless, cruciate ligament repair remains a controversial topic. This is in part due to the unsatisfactory results of early open ACL and PCL repair in the 80 s and 90s [47, 49, 60].

In addition, there is the notion that spontaneous healing of intra-articular ligaments is constrained due to the hostile environment in the knee. The synovial fluid could wash away the blood cloth between the torn ends, complicating the healing process [26, 37]. However, compared to the ACL, the healing capacity of the PCL is less limited and conservative therapies can result in a healed ligament, albeit in a lax or attenuated manner [13, 31, 61].

To explain the self-healing capacity of the PCL, the hypothesis is raised that the position of the PCL combined with the presence of surrounding tissues could prevent the synovial fluid from washing away healing cells. Unlike the ACL, the PCL is not entirely surrounded by the synovial sheet, as the posterior part of the PCL is pressed against the articular capsule. Additionally, the PCL is surrounded by other anatomical structures.

In the current systematic review only two studies, comprising a small number of 25 patients, obtained post-operative MRI and documented successful healing of the repaired PCL [42, 65]. Although not included in this systematic review due to small sample sizes, PCL healing has also been reported on MRI in a number of case reports [8, 15, 21, 40]. Future studies should include post-operative MRI measurements to assess PCL healing, including reporting of MRI parameters and image interpretation methods. Ultimately, quantitative MRI methods would be helpful to objectively assess biomarkers of PCL healing [66].

Good patient selection has been put forward to explain the improved results of modern PCL repair [63]. MRI has made it possible to accurately diagnose PCL tears [16]. However, debate still exists on whether mid-substance tears should be considered a contraindication for PCL repair. It is known that the middle third of the PCL is less vascularized, which could make it more difficult for mid-substance tears to heal [46]. Our systematic review shows that primary PCL repair is mostly performed in proximal tears and to a lesser extent in distal tears. Mid-substance tears are often avoided and sometimes considered a contraindication for PCL repair [63]. On the other hand, in the case series of Otto et al. [42] six of the 14 included patients suffered from a mid-substance tear, all of which were repaired with additional suture augmentation. At final follow-up, healing of the repaired PCL was confirmed based on signal intensity and PCL tissue continuity [42]. In addition, in a recent case report two mid-substance PCL tears were repaired with additional suture augmentation and successful healing was confirmed clinically and on MRI at two years follow-up [21].

This systematic review has limitations. All levels of evidence were included due to the lack of high quality clinical trials. This means that most of the included studies consisted of level IV case series with small sample sizes. The lack of high quality studies can be explained by the low incidence of PCL injuries. Another limitation is the heterogeneity among the studies on PCL repair. Different surgical procedures, consisting of both open and arthroscopic techniques, were used. Follow-up periods also differed considerably between and within the included studies and PCL injuries are often accompanied by a variety of concomitant injuries. Lastly, although the literature search was conducted by two independent reviewers, selection bias remains a potential risk.

## Conclusion

This systematic review indicates that PCL repair can be a safe procedure with an overall failure rate of 5.6%, ranging from 0% to 15.8%. Although PCL repair is gaining attention, it is important to note that current literature is restricted to small case series providing low level evidence with ample risk of bias. More high quality randomized studies are necessary before widespread clinical implementation is warranted.

## Supplementary Information

**Additional file 1.**

## Abbreviations

PCL: Posterior Cruciate Ligament
ACL: Anterior Cruciate Ligament
MLKI: Multiligament Knee Injury
PRISMA: Preferred Reporting Items for Systematic Reviews and Meta-Analyses
LOE: Level of Evidence
IKDC: International Knee Documentation Committee
KOOS: Knee Injury and Osteoarthritis Outcome Scale
VAS: Visual Analogue Scale
MINORS: Methodological Index for Non-randomized Studies
PROMs: Patient-reported Outcome Measures
ROM: Range of Motion
