# Supplementary material for: Posterior cruciate ligament repair seems safe with low failure rates but more high level evidence is needed: a systematic review
Source: J Exp Orthop. 2023 Apr 26;10:49. doi: 10.1186/s40634-023-00605-z (PMC10133428; doi:10.1186/s40634-023-00605-z)
Supplement: Supplementary file 1 — Additional file 1. [file 40634_2023_605_MOESM1_ESM.docx]

Additional file 1

(((("Posterior Cruciate Ligament"[MeSH Terms] OR "PCL"[All Fields] OR (("Posterior Cruciate Ligament"[MeSH Terms] OR ("posterior"[All Fields] AND "cruciate"[All Fields] AND "ligament"[All Fields]) OR "Posterior Cruciate Ligament"[All Fields]) AND ("injurie"[All Fields] OR "injuried"[All Fields] OR "injuries"[MeSH Subheading] OR "injuries"[All Fields] OR "wounds and injuries"[MeSH Terms] OR ("wounds"[All Fields] AND "injuries"[All Fields]) OR "wounds and injuries"[All Fields] OR "injurious"[All Fields] OR "injury s"[All Fields] OR "injuryed"[All Fields] OR "injurys"[All Fields] OR "injury"[All Fields])) OR ("PCL"[All Fields] AND ("lesion"[All Fields] OR "lesion s"[All Fields] OR "lesional"[All Fields] OR "lesions"[All Fields])) OR (("acute"[All Fields] OR "acutely"[All Fields] OR "acutes"[All Fields]) AND "PCL"[All Fields] AND ("lesion"[All Fields] OR "lesion s"[All Fields] OR "lesional"[All Fields] OR "lesions"[All Fields]))) AND ("repairability"[All Fields] OR "repairable"[All Fields] OR "repaire"[All Fields] OR "repaired"[All Fields] OR "repairment"[All Fields] OR "wound healing"[MeSH Terms] OR ("wound"[All Fields] AND "healing"[All Fields]) OR "wound healing"[All Fields] OR "repair"[All Fields] OR "repairing"[All Fields] OR "repairs"[All Fields] OR "primary repair"[All Fields])) NOT ("animal*"[All Fields] OR "not human*"[All Fields] OR "nonhuman*"[All Fields])) NOT ("nano*"[All Fields] OR "polyc*"[All Fields])) NOT ("Child"[All Fields] OR "Children"[All Fields])
